# Supplementary figures and images for: Psychometric properties of the Portuguese version of the National Eye Institute Visual Function Questionnaire-25
Source: PLoS One. 2019 Dec 10;14(12):e0226086. doi: 10.1371/journal.pone.0226086 (PMC6903730; doi:10.1371/journal.pone.0226086)

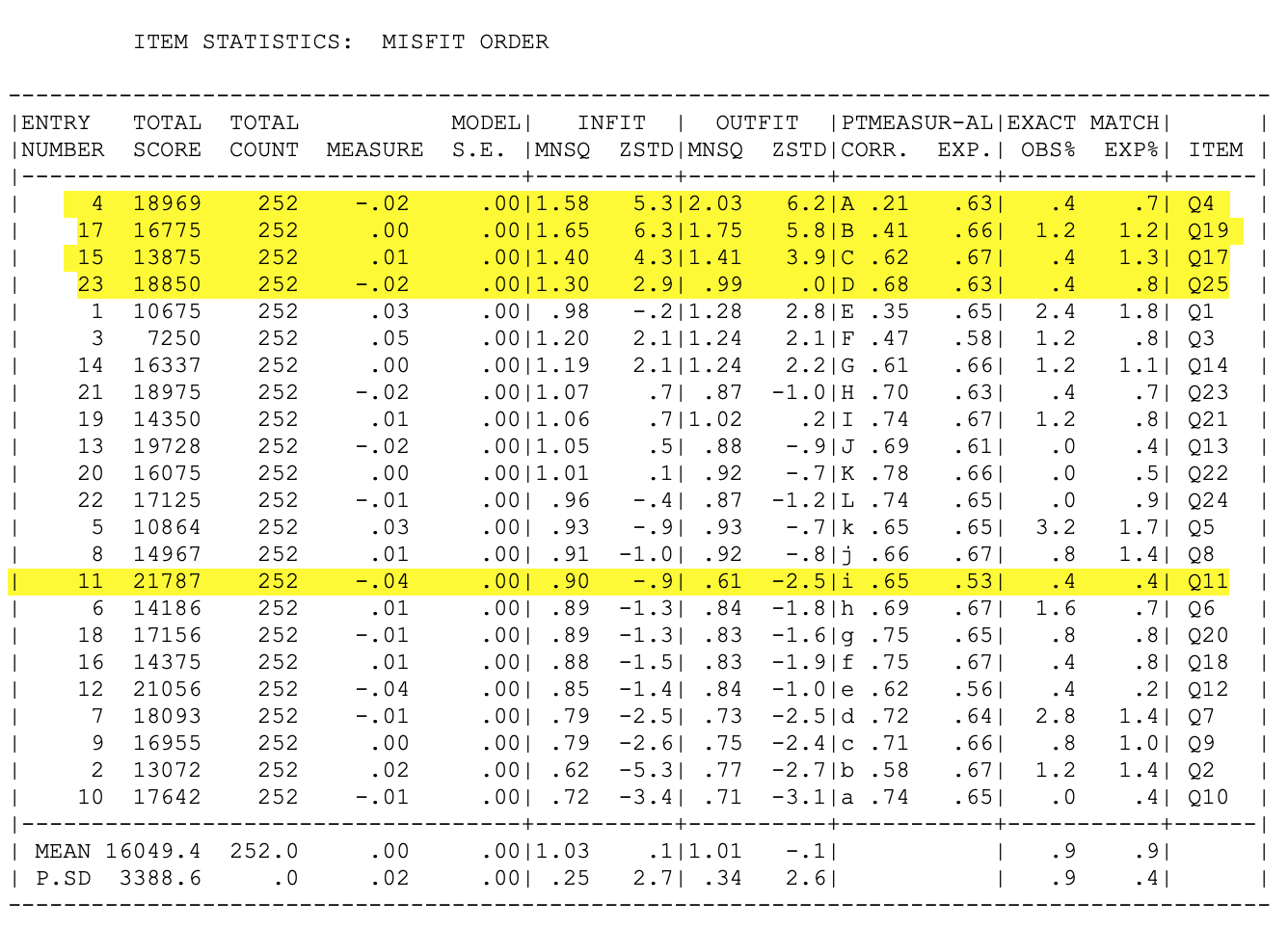

Supplement: S1 Fig — (PNG) [file pone.0226086.s001.png]

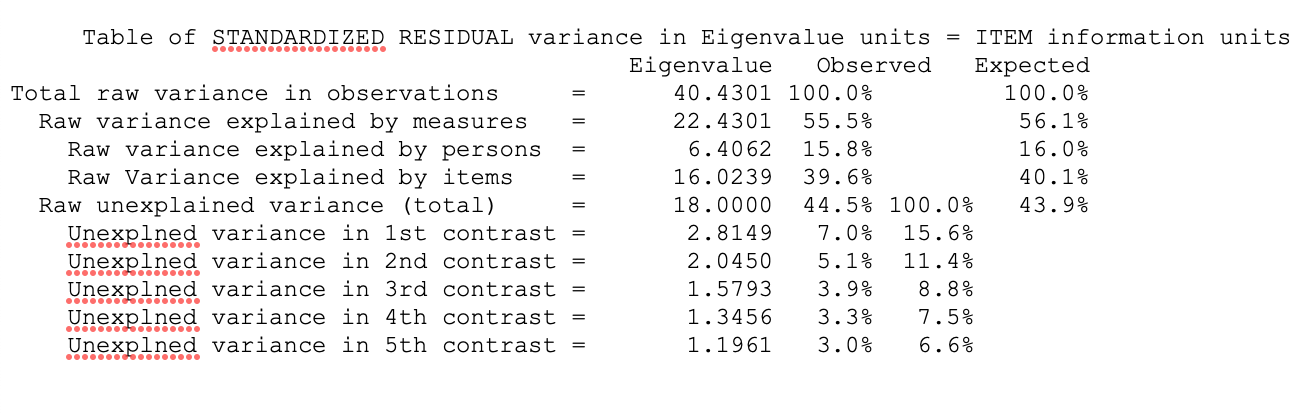

Supplement: S2 Fig — (PNG) [file pone.0226086.s002.png]

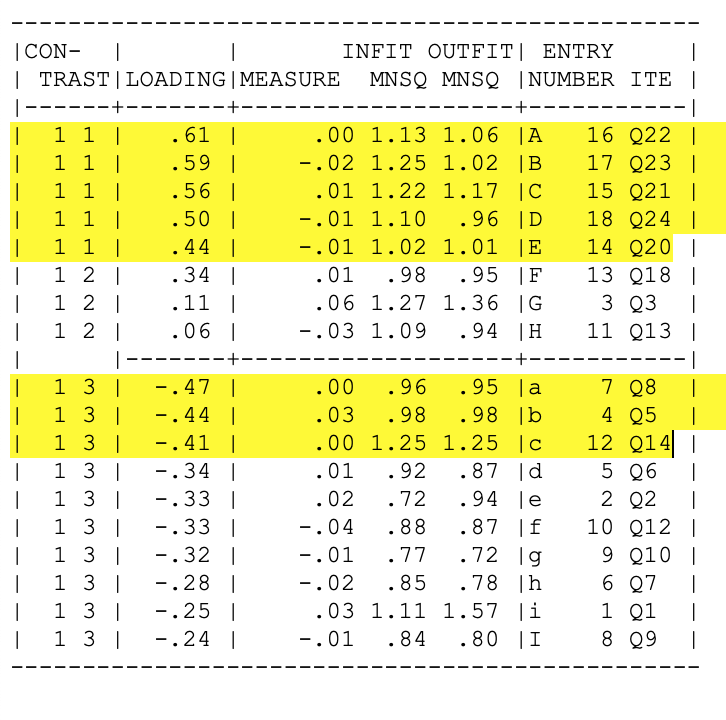

Supplement: S3 Fig — (PNG) [file pone.0226086.s003.png]

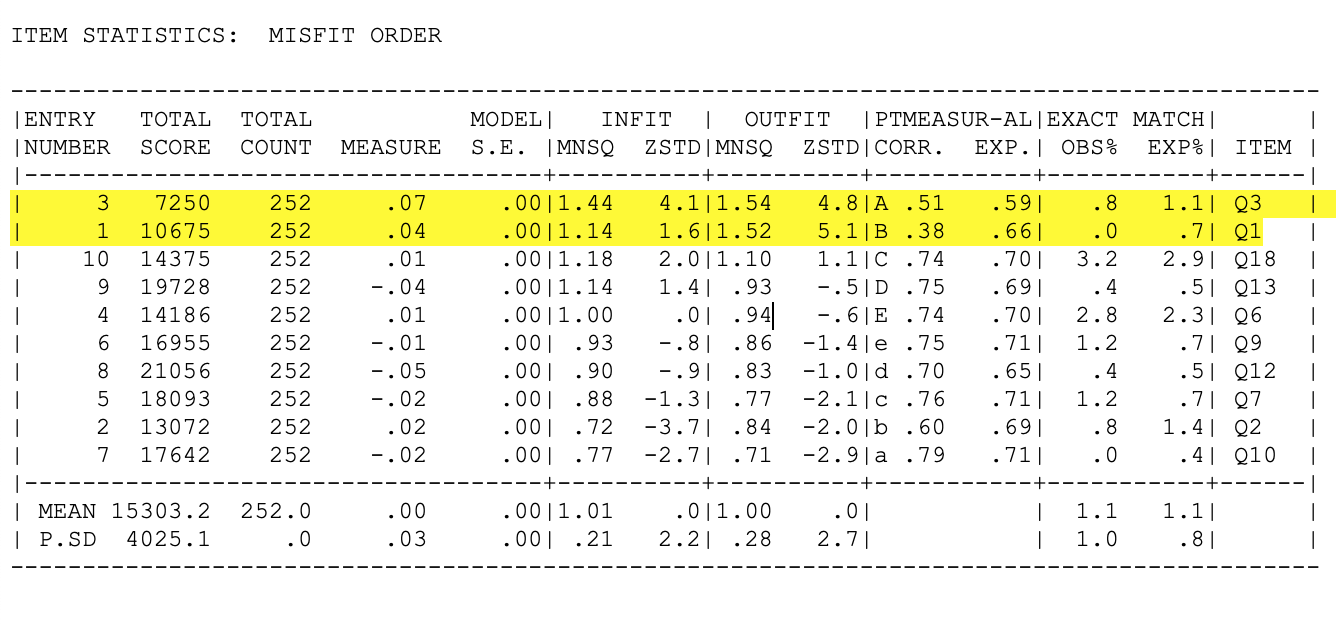

Supplement: S4 Fig — (PNG) [file pone.0226086.s004.png]
