# Supplementary material for: Psychometric properties of the Portuguese version of the National Eye Institute Visual Function Questionnaire-25
Source: PLoS One. 2019 Dec 10;14(12):e0226086. doi: 10.1371/journal.pone.0226086 (PMC6903730; doi:10.1371/journal.pone.0226086)

PLOS ONE

Psychometric Properties of the Portuguese Version of the National Eye Institute Visual Function Questionnaire-25

--Manuscript Draft--

Manuscript Number:	PONE-D-19-19847
Article Type:	Research Article
Full Title:	Psychometric Properties of the Portuguese Version of the National Eye Institute Visual Function Questionnaire-25
Short Title:	Psychometric Properties of the Portuguese Version of the NEI VFQ-25
Corresponding Author:	Ricardo Abe<br>Universidade Estadual de Campinas<br>Campinas, BRAZIL
Keywords:	
Abstract:	Background<br><br>To investigate the psychometric properties of the Brazilian Portuguese version of the National Eye Institute Visual Function Questionnaire (NEI VFQ-25) questionnaire in a group of patients with different eye diseases.<br><br>Methods<br><br>Cross-sectional study. All subjects completed the Portuguese version of the NEI VFQ-25 questionnaire. Another questionnaire containing a survey about clinical and demographics data was also applied. Rasch analysis was used to evaluate the psychometric properties of the NEI VFQ-25.<br><br>Results<br><br>The study included 104 patients with cataract, 65 with glaucoma and 83 with age macular degeneration. Mean age was 70.7 ± 9.9 years, with 143 female (56.7%) and 109 male patients (43.2%). Mean visual acuity was 0.47 and 1.17 logMAR in the better and worse eye, respectively. According to Rasch analysis, four items were found to misfit. Those items belonged to the following subscales: mental health, ocular pain and role limitations. The principal component analysis of the residuals showed that 51.9% of the variance was explained by the principal component. Eight items loaded positively onto the first contrast with a correlation higher than 0.4. These items belonged to the following subscales: general health, mental health, role limitations and dependency. After excluding those items, we were able to isolate items from the NEI VFQ-25, related only to a visual functioning component. Finally, the principal component analysis from residuals of this revised version of the NEI VFQ-25 (items related to visual function) showed that the principal component explained 61.2% of the variance, showing no evidence of multidimensionality.<br><br>Conclusions<br><br>The Portuguese version of the NEI VFQ-25 is not a unidimensional instrument. We were able to find items that belong to a different trait, possible related to a socio-emotional component. Thus, in order to obtain psychometrically valid constructs, both the visual functioning and socio-emotional components should be analyzed separately.
Order of Authors:	Ricardo Y Abe
	Felipe A. Medeiros
	Milton Agrizzi Davi
	Cecília Gonçalves
	Matheus Bittencourt

Powered by Editorial Manager® and ProduXion Manager® from Aries Systems Corporation

	Alicia Buffoni Roque
	Júlia Boccato
	Vital Paulino Costa
	José Paulo Cabral Vasconcellos
**Additional Information:**	
**Question**	**Response**
**Financial Disclosure**<br><br>Enter a financial disclosure statement that describes the sources of funding for the work included in this submission. Review the submission guidelines for detailed requirements. View published research articles from *PLOS ONE* for specific examples.<br><br>This statement is required for submission and **will appear in the published article** if the submission is accepted. Please make sure it is accurate.<br><br>**Unfunded studies**<br>Enter: *The author(s) received no specific funding for this work.*<br><br>**Funded studies**<br>Enter a statement with the following details:<br>• Initials of the authors who received each award<br>• Grant numbers awarded to each author<br>• The full name of each funder<br>• URL of each funder website<br>• Did the sponsors or funders play any role in the study design, data collection and analysis, decision to publish, or preparation of the manuscript?<br>• **NO** - Include this sentence at the end of your statement: *The funders had no role in study design, data collection and analysis, decision to publish, or preparation of the manuscript.*<br>• **YES** - Specify the role(s) played.<br><br>* typeset	NO
**Competing Interests**<br><br>Use the instructions below to enter a competing interest statement for this submission. On behalf of all authors, disclose any competing interests that	NO authors have competing interests

could be perceived to bias this work—acknowledging all financial support and any other relevant financial or non-financial competing interests.<br><br>This statement **will appear in the published article** if the submission is accepted. Please make sure it is accurate. View published research articles from *PLOS ONE* for specific examples.<br><br>**NO authors have competing interests**<br><br>Enter: *The authors have declared that no competing interests exist.*<br><br>**Authors with competing interests**<br><br>Enter competing interest details beginning with this statement:<br><br>*I have read the journal's policy and the authors of this manuscript have the following competing interests: [insert competing interests here]*<br><br>* typeset	
**Ethics Statement**<br><br>Enter an ethics statement for this submission. This statement is required if the study involved:<br><br>• Human participants<br>• Human specimens or tissue<br>• Vertebrate animals or cephalopods<br>• Vertebrate embryos or tissues<br>• Field research<br><br>Write "N/A" if the submission does not require an ethics statement.<br><br>General guidance is provided below. Consult the submission guidelines for detailed instructions. **Make sure that all information entered here is included in the Methods section of the manuscript.**	This study was approved by the Ethics Committee of the University of Campinas and adhered to the tenets of the Declaration of Helsinki.<br><br>CAAE: 47862215.0.0000.5404<br><br>A written consent form was obtained from all patients.

Powered by Editorial Manager® and ProduXion Manager® from Aries Systems Corporation

**Format for specific study types**<br><br>**Human Subject Research (involving human participants and/or tissue)**<br>• Give the name of the institutional review board or ethics committee that approved the study<br>• Include the approval number and/or a statement indicating approval of this research<br>• Indicate the form of consent obtained (written/oral) or the reason that consent was not obtained (e.g. the data were analyzed anonymously)<br><br>**Animal Research (involving vertebrate animals, embryos or tissues)**<br>• Provide the name of the Institutional Animal Care and Use Committee (IACUC) or other relevant ethics board that reviewed the study protocol, and indicate whether they approved this research or granted a formal waiver of ethical approval<br>• Include an approval number if one was obtained<br>• If the study involved *non-human primates*, add *additional details* about animal welfare and steps taken to ameliorate suffering<br>• If anesthesia, euthanasia, or any kind of animal sacrifice is part of the study, include briefly which substances and/or methods were applied<br><br>**Field Research**<br><br>Include the following details if this study involves the collection of plant, animal, or other materials from a natural setting:<br>• Field permit number<br>• Name of the institution or relevant body that granted permission	
**Data Availability**<br><br>Authors are required to make all data underlying the findings described fully available, without restriction, and from the time of publication. PLOS allows rare exceptions to address legal and ethical concerns. See the PLOS Data Policy and FAQ for detailed information.	Yes - all data are fully available without restriction

A Data Availability Statement describing where the data can be found is required at submission. Your answers to this question constitute the Data Availability Statement and **will be published in the article**, if accepted.<br><br>**Important:** Stating 'data available on request from the author' is not sufficient. If your data are only available upon request, select 'No' for the first question and explain your exceptional situation in the text box.<br><br>Do the authors confirm that all data underlying the findings described in their manuscript are fully available without restriction?	
**Describe where the data may be found in full sentences. If you are copying our sample text, replace any instances of XXX with the appropriate details.**<br><br>• If the data are **held or will be held in a public repository**, include URLs, accession numbers or DOIs. If this information will only be available after acceptance, indicate this by ticking the box below. For example: *All XXX files are available from the XXX database (accession number(s) XXX, XXX.).*<br>• If the data are all contained **within the manuscript and/or Supporting Information files**, enter the following: *All relevant data are within the manuscript and its Supporting Information files.*<br>• If neither of these applies but you are able to provide **details of access elsewhere**, with or without limitations, please do so. For example:<br><br>*Data cannot be shared publicly because of [XXX]. Data are available from the XXX Institutional Data Access / Ethics Committee (contact via XXX) for researchers who meet the criteria for access to confidential data.*<br><br>*The data underlying the results presented in the study are available from (include the name of the third party*	The data underlying the results presented in the study are available from (Ricardo Yuji Abe, ricardoabe85@yahoo.com.br)

and contact information or URL).<br>• This text is appropriate if the data are owned by a third party and authors do not have permission to share the data.<br><br>* typeset	
Additional data availability information:	Tick here if your circumstances are not covered by the questions above and you need the journal's help to make your data available.

Click here to download Manuscript Manuscript - 14 july 2019.docx

Psychometric Properties of the Portuguese Version of the National Eye Institute Visual Function Questionnaire-25

Ricardo Y. Abe[1¶], Felipe A. Medeiros [2¶], Milton Agrizzi Davi[1¶], Cecília Gonçalves[1¶], Matheus Bittencourt[1¶], Alicia Buffoni Roque[1¶], Júlia Boccato[1¶], Vital Paulino Costa[1¶], José Paulo Cabral Vasconcellos[1¶]

1. Department of Ophthalmology, University of Campinas, Campinas, Brazil.
2. Department of Ophthalmology, Duke University, Durham, North Carolina, United States

Corresponding Author:

ricardoabe85@yahoo.com.br (RYA)

¶These authors contributed equally to this work.

ABSTRACT

Background: To investigate the psychometric properties of the Brazilian Portuguese version of the National Eye Institute Visual Function Questionnaire (NEI VFQ-25) questionnaire in a group of patients with different eye diseases.

Methods: Cross-sectional study. All subjects completed the Portuguese version of the NEI VFQ-25 questionnaire. Another questionnaire containing a survey about clinical and demographics data was also applied. Rasch analysis was used to evaluate the psychometric properties of the NEI VFQ-25.

Results: The study included 104 patients with cataract, 65 with glaucoma and 83 with age macular degeneration. Mean age was 70.7 ± 9.9 years, with 143 female (56.7%) and 109 male patients (43.2%). Mean visual acuity was 0.47 and 1.17 logMAR in the better and worse eye, respectively. According to Rasch analysis, four items were found to misfit. Those items belonged to the following subscales: mental health, ocular pain and role limitations. The principal component analysis of the residuals showed that 51.9% of the variance was explained by the principal component. Eight items loaded positively onto the first contrast with a correlation higher than 0.4. These items belonged to the following subscales: general health, mental health, role limitations and dependency. After excluding those items, we were able to isolate items from the NEI VFQ-25, related only to a visual functioning component. Finally, the principal component analysis from residuals of this revised version of the NEI VFQ-25 (items related to visual function)

showed that the principal component explained 61.2% of the variance, showing no evidence of multidimensionality.

Conclusions: The Portuguese version of the NEI VFQ-25 is not a unidimensional instrument. We were able to find items that belong to a different trait, possible related to a socio-emotional component. Thus, in order to obtain psychometrically valid constructs, both the visual functioning and socio-emotional components should be analyzed separately.

Keywords: Glaucoma; Quality of life; National Eye Institute Visual Function Questionnaire; NEI VFQ-25; Rasch Analysis

INTRODUCTION

Quality of life (QoL) is a broad-ranging concept affected by an individual's physical health, psychological state, level of independence and social relationships.[1, 2] Within physical health, sense of sight is crucial to perform many routine daily activities. Therefore, changes in visual status can lead to functional impairment affecting directly QoL. Conventional clinical measures such as visual acuity, visual field assessment and fundus imaging may not fully capture the impact of disability related to eye diseases. Thus measurements of health-related QoL have been used to track outcomes for many eye diseases.[3, 4] Even though many so-called health-related QoL questionnaires can measure only a self-perceived health status, the importance of evaluating the outcomes of health care from the standpoint of the patient is now widely recognized.[5, 6]

Within patient-reported questionnaires, the National Eye Institute Visual Function Questionnaire (NEI VFQ-25) has been frequently used to assess QoL in ophthalmology research.[7-10] This questionnaire contains a set of 25 questions in 12 subscales designed to assess the dimensions of self-reported vision-target health status that are relevant for subjects with chronic eye diseases.[7] Items in the questionnaire require the subject to provide a response based on a Likert scale attributing linear scores to each response and then summing up the scores for all different questions to obtain a single composite score. However, in order for a composite score to be meaningful is essential that all questions included in the scoring contribute to the measurement of a single underlying construct. [3, 11] For example, for the NEI VFQ-25 responses to be represented by a single score, its questions should all be measuring the same latent construct of visual functioning.

Rasch analysis is a method that can be used to investigate psychometric properties of questionnaires, such as dimensionality and reliability. Massof et al administered some items from the 52-NEI-VFQ to patients with low vision, applying Rasch model to estimate interval measurement scales from ordinal responses to items.[11] They found that Rasch analysis can offer an alternative approach to traditional scoring methods enabling one to estimate the latent variable of interest (visual function) and assess the performance of each item as a contributor to the final measurement. In a subsequent work, Marella and colleagues have suggested that the NEI VFQ-25 questionnaire does not seem to be unidimensional, and that the questionnaire items may actually be measuring two different underlying constructs, one related to visual functioning and another to socio-emotional status. [12, 13] This is important, as it would indicate that a single composite score is not appropriate to represent responses from this questionnaire. [12, 13] In addition to dimensionality, Rasch analysis can provide information about appropriateness of the response categories, measurement precision, and item fit to the construct.[14] Rasch analysis of the English version of the NEI VFQ-25 has also suggested that the subscales represented on the questionnaire would not be valid in their current format.[15]

As a widely used instrument to assess vision-related QoL, the NEI VFQ-25 has been translated into several different languages. When a questionnaire is translated into a new language, a linguistic validation is necessary but not sufficient unless the psychometric characteristics have been verified. Simao et al. introduced the Brazilian Portuguese version of the NEI VFQ-25 in 2008 and reported psychometrics properties comparable to the American original version.[16] However, the work of Simao et al

reported only measures such as Cronbach's alpha and correlations among subscales. The reason Rasch analysis is well suited for demonstrating a translated version of an existing questionnaire has comparable items to the original is its sensitivity to differences in item difficulty.[17] Cronbach's alpha, for example, is insensitive to this, and can provide the same value for two questionnaires whose items differ in levels of difficulty. Thus, a proper validation of the Portuguese translation of the questionnaire would also benefit from a method such as Rasch analysis to better assess dimensionality and validity. To the best of our knowledge no study has yet applied the Rasch Analysis to the Brazilian Portuguese version of the NEI VFQ-25 questionnaire. Thus, the purpose of current study is to investigate the psychometric properties of NEI VFQ-25 using Rasch analysis in a population of Brazilian patients with a variety of eye diseases.

MATERIALS AND METHODS

This was a cross sectional study, evaluating patients with glaucoma, cataract and age-related macular degeneration (AMD) from the Hospital das Clínicas – University of Campinas – Brazil. This study was approved by the Ethics Committee of the University of Campinas and adhered to the tenets of the Declaration of Helsinki. Subjects underwent a comprehensive ophthalmic examination, including Snellen best corrected visual acuity, slit-lamp biomicroscopy, intraocular pressure (IOP) measurement using Goldmann applanation tonometry, gonioscopy and dilated fundoscopy examination using a 78-diopter lens. Subjects underwent standard automated perimetry (SAP), using the 24-2 Swedish Interactive Threshold Algorithm (SITA) Standard (Humphrey, Carl Zeiss

Meditec, Inc., Dublin, CA, USA) and also retinal imaging with nonmyd WX^{3D} (Kowa, Japan).

Three different groups were investigated. Glaucoma patients were required to have repeatable (at least 2 consecutive) abnormal SAP results with corresponding glaucomatous optic nerve damage in at least one eye. An abnormal SAP result was defined as a pattern standard deviation with $P < 0.05$, and/or Glaucoma Hemifield Test results outside normal limits. Cataracts were classified according to the Lens Opacities Classification System III (LOCS III) based on findings from slit lamp examination.[18] For AMD, we applied the Clinical Age-Related Maculopathy Staging system, which divides patients into 5 mutually exclusive categories based on slit-lamp assessment of drusen, retinal pigment epithelial irregularities, geographic atrophy, retinal pigment epithelial detachment, and choroidal neovascularization.[19, 20]

NEI VFQ-25 Questionnaire

Vision-related QoL was assessed by a Brazilian Portuguese version of the NEI VFQ-25 questionnaire.[7] This version was developed by Simao et al in 2008 and was initially tested for in a set of ophthalmic patients and healthy controls.[16] The NEI VFQ-25 consists of 25 questions measuring overall vision, difficulty with near-vision and distance activities, ocular pain, driving difficulties, limitations with peripheral vision and color vision, social functioning, role limitations, dependency and mental health symptoms. Rasch analysis was performed to obtain final estimates of “person measures,” which can be used to express where each respondent falls on a linear scale representing

the degree of impairment as measured by the NEI VFQ-25.[21, 22] Rasch analysis was performed using Andrich rating-scale models to obtain the estimates of the required ability of each item, perceived ability of each subject, and the thresholds for each response category.[14] The unit of those estimated measures is called a logit (log-odds unit), which is calculated as the log-odds ratio of the probability that a participant will select a particular rating category in an item over 1 minus the same probability. The logit values place patients according to their abilities and items according to their difficulties on the same linear interval scale. [23]

Person and item measures were examined for fit to the Rasch model using infit and outfit item fit statistics.[8] To test the hypothesis that the NEI VFQ- 25 measures a single underlying construct, we initially evaluated the fit statistics, which were recorded as mean square standardized residuals (MNSQ); The fit of the Rasch model was evaluated with the infit and outfit statistics. Values between 0.7 and 1.3 are considered acceptable for MNSQ values of infit and outfit.[24] After checking fit statistics, we conducted a principal components analysis of the residuals (difference between the observed and expected responses).[25] Data are considered unidimensional if most of the variance is explained by the principal component and there is no significant explanation of the residual variance by the contrasts to the principal component. In general, to be considered unidimensional, the variance of the principal component should be >60%.[8, 25] Furthermore, the unexplained variance by the contrasts should be <2 Eingenvalue units.[25]

We also evaluated differential item functioning, which assesses whether the items have different meanings for different groups in the sample. The raw differences in item

calibration between groups were examined to identify differential item functioning. The differential item functioning was considered absent if it was less than 0.50 logits, minimal but probably inconsequential if it ranged between 0.50 and 1.0 logits, and notable if it was >1.0 logit.[12, 26]

The person separation index is the ratio of the variance in the person measures for the sample to the average error in estimating these measures. It is a measure of how broadly the persons could be distinguished into statistically distinct levels. The person separation reliability coefficient describes the reliability of the scale to discriminate between the persons of different abilities.[12] A person separation index of ≥ 2.0 or a reliability value of ≥ 0.8 represents the minimum acceptable level of separation.[12, 24]

Demographic, Clinical and Socio-economic Variables

Socio-economic questionnaires were also administered along with the NEI VFQ-25 to all patients. These questionnaires contained a survey about demographics, history of ocular and medical conditions, marital status, degree of education and income. For comorbidities, we investigated the presence or history of the following conditions: diabetes mellitus, arthritis, high blood pressure, heart disease, depression, asthma, and cancers. A simple summation score was used to create a comorbidity index.[27] As these variables could potentially affect patient perceptions about vision-related QOL, they were included as covariates factors to investigate their association with the final Rasch-calibrated NEI VFQ-25 scores. These variables were categorized for inclusion in the univariate and multivariate models as race (African American [yes/no]), employment (yes/no), marital status (married [yes/no]), degree of education (at least high school

degree [yes/no]) and income (less than $25,000/year [yes/no]). Visual acuity was measured using an Early Treatment Diabetic Retinopathy (ETDRS) chart and logMAR measurements were used in the analyses evaluating better and worse eye.[28] For patients with visual acuity measures of "counting fingers" (CF), "hand motion", "light perception" and "no light perception" (NLP), we converted into quantitative measurements such as logMAR, as suggested by Schulze-Bonsel. [28]

Statistical Analysis

Descriptive statistics included mean and standard deviation for normally distributed variables. We investigated the relationship between final Rasch-calibrated NEI VFQ-25 scores with socioeconomic and clinical variables (gender, race, education, income, marital status, visual acuity in logMAR, presence of low vision and mean deviation from standard automated perimetry) using a linear regression model. Variables with P value < 0.2 were included in the final multivariable linear regression model. Statistical analyses were performed using Winsteps 3.81.0 (Chicago, IL) and STATA v. 13 (StataCorp, College Station, TX). The alpha level (type I error) was set at 0.05.

RESULTS

The study included 104 patients with cataract, 6[illegible] with glaucoma and 8[illegible] with AMD. Table 1 presents demographic variables of the studied population. Mean age was 70.7 ± 9.9 years, with 143 female (56.7%) and 109 male patients (43.2%). Most of them were retired (74%). Table 2 describes the clinical variables of the patients. Mean visual acuity in the better eye was 0.47 logMAR and 1.17 logMAR in worse eye. There were 62

patients (24.6%) with low vision (counting fingers, hand motion, light perception or loss of light perception in one or both eyes).

Table 1. Demographic characteristics of all patients included in the study.

Parameters	Total subjects (n = 252)
**Age (years)**	
**Mean ± SD**	70.7± 9.9
**Range**	30 to 103
**Gender, n (%)**	
**Male**	109 (43.2%)
**Female**	143 (56.7%)
**Race, n (%)**	
**Caucasian**	204 (81.2%)
**African-American**	44 (17.5%)
**Job status (%)**	
**Employed**	44 (18.1%)
**Unnenployed**	19 (7.8%)
**Retired**	180 (74.0%)
**Marital status (%)**	
**Married**	119 (69.5%)
**Single**	25 (14.6%)
**Widowed**	14 (8.1%)
**Divorced**	13 (7.6%)
**Education (%)**	
**Illiterate**	2 (0.8%)
**Elementary school**	137 (56.6%)
**High school degree**	58 (23.9%)
**College degree**	8 (3.2%)
**Income per month (%)**	
**Lower than US$414.00**	81 (48.2%)
**Between US$414.00 and US$2,073.00**	68 (40.4%)
**Between US$2,073.00 and US$4,147.00**	17 (10.1%)
**Higher than US$4,147.00**	2 (1.1%)
**Comorbidity Index (%)**	
**Zero**	71 (28.9%)
**One**	109 (44.4%)
**Two**	58 (23.6%)
**Three**	6 (2.4%)

Table 2. Clinical characteristics of all patients included in the study.

Parameters	Total subjects (n = 252)
LogMar Visual acuity (better eye)	
Mean ± SD	0.47 ± 0.39
LogMar Visual acuity (worse eye)	
Mean ± SD	1.17 ± 0.74
SAP MD from glaucoma (better eye) (dB)	
Mean ± SD	-4.26 ± 3.85
SAP MD OS from glaucoma (worse eye) (dB)	
Mean ± SD	-10.77 ± 9.38
Low vision, n (%)	62 (24.6%)

SD: standard deviation; SAP: standard automated perimetry; MD: mean deviation; OD: right eye; OS: left eye.

Rasch Analysis

Results of Rasch analysis are shown in Table 3. Four items (Q4, Q19, Q24 and Q25) were found to misfit (from subscales: general health, mental health, ocular pain and role limitations) with infit and/or outfit mean scores >1.3. Fig 1 shows a scatterplot with the items with misfit. Principal components analyses of the residuals from Rasch analysis can also be used to check the assumption of unidimensionality.[29] In order to determine whether the assumption of unidimensionality is valid, the variance explained by the Rasch factor (the underlying construct) should be 4 times greater than that of the first principal component in the residuals and the variance explained by the Rasch factor should be greater than 60%.[30]

Fig 1. Scatterplot of infit versus outfit statistics for item measures estimated from responses to items in the National Eye Institute Visual Function Questionnaire (NEI VFQ-25). The box bounds the 1.3 unacceptable limits, highlighting the misfitting items inside the box.

Table 3. Fit Statistics using Rasch Analysis with respective Items and Subscales from National Eye Institute Visual Function Questionnaire (NEI VFQ-25).

Questions	Items	Subscales	Measure	Infit MNSQ	Outfit MNSQ
Q1	General health	General health	-0.19	1.09	1.12
Q2	General vision	General vision	-0.30	0.62	0.77
Q3	Worry about eyesight	Mental health	-0.46	1.05	1.21
Q4	Pain around eyes	Ocular pain	0.41	1.58	2.03
Q5	Reading normal newsprint	Near vision	-0.22	0.93	0.93
Q6	Seeing well up close	Near vision	0.07	0.89	0.84
Q7	Finding objects on crowded shelf	Near vision	0.40	0.79	0.73
Q8	Street signs	Distance vision	0.13	0.91	0.92
Q9	Going downstairs at night	Distance vision	0.30	0.79	0.75
Q10	Seeing objects off to side	Peripheral vision	0.36	0.72	0.68
Q11	Seeing how people react	Social function	0.91	0.90	0.61
Q12	Matching clothes	Color vision	0.79	0.85	0.84
Q13	Visiting others	Social function	0.56	1.05	0.88
Q14	Going out to movies/plays	Distance vision	0.26	1.19	1.24
Q17	Accomplish less	Role limitations	-0.37	1.40	1.41
Q18	Limited endurance	Role limitations	-0.42	0.88	0.83
Q19	Amount of time in pain	Ocular pain	-0.18	1.65	1.75
Q20	Stay home most of the time	Dependency	-0.32	0.89	0.83
Q21	Frustrated	Mental health	-0.51	1.06	1.02
Q22	No control	Mental health	-0.32	1.01	0.92
Q23	Rely too much on others' words	Dependency	-0.51	1.07	0.87
Q24	Need much help from others	Dependency	-0.40	0.96	0.87
Q25	Embarrassment	Mental health	-0.51	1.30	0.99

MNSQ: mean square

For the current work, the principal component analysis of the residuals showed that the variance explained by the principal component was comparable for empirical calculation (50.9%) and by the model (51.9%). This suggests that the questionnaire was not unidimensional. Moreover, the unexplained variance explained by the first contrast was 3.38 eigenvalue units and the second contrast was 2.56 eigenvalue units with no further contrasts exceeding 2.0 eigenvalue units. These findings suggested the presence of a second dimension in the scale. Eight items loaded (correlation>0.4) positively onto the first contrast and belonged to: general health (Q1), mental health (Q3, Q21and Q22), role limitations (Q17 and Q18) and dependency (Q20 and Q23). This suggests that these eight items cannot be grouped with other items in the scale to measure a single latent trait (visual functioning). These items are probably related to a social-emotional component as previously stated by Marella et al.[12] Of note, in the current sample, 187 patients (74.4%) answered that they were not currently driving (Q15). Within this group, 158 patients (84.5%) reported that they never had driven (Q15a). Therefore questions related to driving were not assessed in the Rasch Analysis due to missing data.

Differential item functioning was tested for some of the variables from Table 1 and Table 2, such as: age, gender, race, job status, marital status, education, level of income, low vision and type of eye disease (cataract, glaucoma and AMD). There was no differential item functioning for any of the variables mentioned. These results suggest that items could be interpreted similarly across subgroups of the sample.

After excluding items that were considered misfitted (Q4, Q19, Q24 and Q25) and also those items with high loadings on the principal component analysis of the residuals, such as: general health (Q1), mental health (Q3, Q21 and Q22), role limitations (Q18)

and dependency (Q20 and Q23), we were able to isolate items from the NEI VFQ-25, related only to the visual function component. According to Table 4, no items were misfitted. We also performed a principal component analysis of the residuals of the revised version of the NEI VFQ-25 (items related to visual function). The final variance of the principal component was 61.2% and the unexplained variance by the contrasts is 1.64 eingenvalue units, showing no evidence of multidimensionality (Table 5). The mean (± SD) of the person measures was -3.02 ± 1.09 logits. In Fig 2 we showed the Wright item-person maps of the revised version of the NEI VFQ-25 (only items related to visual function). The separation index for person measures was 2.44, with reliability of 0.86. We also reported the psychometric properties of the socioemotional component of the NEI VFQ-25 (Table 5).

Fig. 2. Wright item-person maps related to visual function of the revised version of the National Eye Institute Visual Function Questionnaire (NEI VFQ-25). The left-hand column locates the person ability measures along the variable. The right-hand column locates the item difficulty measures along the variable.

Table 4. Fit Statistics from Rasch Analysis with respective Items and Subscales using the only item related to visual function of the National Eye Institute Visual Function Questionnaire (NEI VFQ-25).

Questions	Items	Subscales	Measure	Infit MNSQ	Outfit MNSQ
Q2	General vision	General vision	-1.13	0.77	0.92
Q5	Reading normal newsprint	Near vision	-1.06	1.10	1.08
Q6	Seeing well up close	Near vision	-0.47	0.99	0.99
Q7	Finding objects on crowded shelf	Near vision	0.23	0.90	0.84
Q8	Street signs	Distance vision	-0.34	0.98	0.96
Q9	Going downstairs at night	Distance vision	0.02	0.90	1.00
Q10	Seeing objects off to side	Peripheral vision	0.14	0.75	0.71
Q11	Seeing how people react	Social function	1.17	1.00	0.88
Q12	Matching clothes	Color vision	0.97	0.88	1.04
Q13	Visiting others	Social function	0.54	1.25	1.15
Q14	Going out to movies/plays	Distance vision	-0.07	1.21	1.22

MNSQ: mean square

Table 5. Rasch Analysis Fit Statistics of the Visual Function and Socioemotional Components from the National Eye Institute Visual Function Questionnaire (NEI VFQ 25).

Components	Visual Function	Socioemotional
Items in Scale (n)	11	8
Misfitting Items (n)	None	2 (Q1 and Q3)
Person Separation Index	2.44	1.34
Person Separation Reliability (logits)	0.85	0.64
Mean Person Measure (logits)	-3.02	-1.12
Final variance of Principal Component (%)	61.20	64.1

We also investigated the association between demographic and clinical variables with the final scores of the revised version of the NEI VFQ-25. Within the clinical and demographic variables, there was a statistical relationship with the Rasch-calibrated scores in NEI VFQ-25 for the following variables in univariable models: visual acuity in the better eye (P<0.001), visual acuity in the worse eye (P<0.001), patients with low vision (P<0.001), gender (P<0.001), marital status (P=0.001), employment status (P=0.019), education level (P<0.001) and comorbidity index (P=0.003). In a multivariable analysis, only 2 variables remained statistically significant: visual acuity in

the better eye (P<0.001) and education level (P=0.002).

DISCUSSION

In the current study, we investigated the psychometric properties of the Brazilian Portuguese version of the NEI VFQ-25 using Rasch analysis. Our results showed that the NEI VFQ-25 does not seem to be an unidimensional instrument; that is, it does not measure a single latent construct (quality of life related to visual function).[11] Although most items on the NEI VFQ-25 tap the construct of visual functioning, our results indicated that other items belonged to a different construct, namely socio-emotional component, corroborating findings from previous studies.[12, 13]

Unidimensionality of an instrument can be assessed by examining the fit statistics and principal component analysis of the residuals. Ideally items should have MNSQ values between 0.7 and 1.3. Items with MNSQ lower than 0.7 suggest a high level of predictability in the responses, indicating redundancy, [24] whereas values higher than 1.3 show an unacceptable level of noise in the responses. According to Table 3, four items (Q4, Q19, Q24 and Q25) were found to misfit. Those items belonged to the subscales of mental health, ocular pain and role limitations. In Figure 1 we can observe a scatterplot with the items that we considered misfitted.

In addition to that, we also need to exam the principal component analysis of the residuals as a second test for unidimensionality. A high level of variance accounted for by the principal component leads to a low likelihood of additional components; a variance of 60% or greater is considered good. In the current study, the principal component analysis of the residuals showed that the variance explained by the principal

component was 51.9%. Moreover, the unexplained variance explained by the first contrast was 3.38 eigenvalue units. The first contrast in the residuals reports whether there are patterns within variance that are unexplained by the principal component, which suggests a second construct is being measured.[13] According to previous studies, the current study applied the criterion that the contrast should have an eigenvalue higher than 2.0 to be considered evidence of a second construct because this would be greater than the magnitude seen with random data. Thus, our analysis showing the first contrast with a 3.38 eigenvalue units, suggests that the Brazilian Portuguese version of the NEI VFQ-25 was not unidimensional.

The loading of items onto the contrasts allows identification of which items tap different constructs. In our analysis, eight items loaded positively onto the first contrast with a correlation higher than 0.4. These items belonged to the following subscales: general health (Q1), mental health (Q3, Q21 and Q22), role limitations (Q17 and Q18) and dependency (Q20 and Q23). This suggests that these eight items cannot be grouped with other items in the scale to measure a single latent construct, such as QoL related to visual function.

We were able to isolate items from the NEI VFQ-25, related only to a visual function component, after excluding items that were considered misfitted and also those items with high loadings on the principal component analysis of the residuals. When we re-examined the fit statistics of this revised version of the NEI VFQ-25, no items were misfitted (Table 4). Moreover, the final variance of the principal component was 61.2% and the unexplained variance by the first contrast was 1.64 eingenvalue units (Table 5).

These results suggest that this revised version of the NEI VFQ-25 showed no evidence of multidimensionality.

Simao et al used a “Factor analysis” and concluded that almost all subscales of NEI VFQ-25 belong to the same underlying dimension. However, careful analysis of their data suggests some evidence of multidimensionality.[16] For example, they showed that most of the subscales from the Portuguese version of the NEI VFQ-25 were influenced by central vision correlated with the first factor, while the “General vision”, “Ocular pain” and “Peripheral vision” subscales were included in a second factor.[16] We were able to find a second construct more related to a socio-emotional component formed by subscales such as: “General health”, “mental health”, “role limitations” and “dependency”, in contrast to central and peripheral vision constructs as highlighted in the previous study. This difference might be due to application of different types of analysis (Rasch as opposed to Factor Analysis). When evaluating an instrument with the Rasch model, more fundamental evidence may be provided to justify the use of scale scores on an interval level. Distances on the scales developed by the Factor Analysis approach are interpreted as equal over the full range of the scale.[31] The scale is treated as an interval scale based on ordinal level item scoring. In fact, Pesudovs et al investigated the psychometric properties of the NEI VFQ-25 with Rasch analysis in a group of patients with cataract and found that several subscales were not psychometrically sound. They concluded that the NEI VFQ-25 as an overall measure was flawed by multidimensionality.[13] In addition, Marella et al performed a similar investigation with a group of low vision patients and also found that the NEI VFQ-25 is a better performing

instrument when divided into two different scales, corroborating the findings of our study. [12]

Another important characteristic of a good instrument is that items function similarly for persons at the same level of ability. Differential item functioning was tested for the following variables: age, sex, race, job status, marital status, education, level of income, low vision and type of eye disease (cataract, glaucoma and AMD). Differential item functioning occurs when subgroups of people with comparable levels of ability respond differently to an item, which implies a response to some characteristic other than item difficulty. We were not able to find evidence of differential item functioning for any of the variables mentioned. Thus, our results suggest that items from the Portuguese version of the NEI VFQ-25 could be interpreted similarly across subgroups of the sample, including different eye diseases, such as cataract, glaucoma and AMD.

We found that worse visual acuity and patients with lower education level had lower Rasch-calibrated NEI VFQ-25 scores. Even though patients with AMD had lower Rasch-calibrated scores of NEI VFQ-25 compared to cataract and glaucoma patients, when adjusting for visual acuity, the correlation with different types of eye disease in the multivariable analysis was not statistically significant, implying that visual acuity may be a better predictor for vision related QoL in comparison to the underlying cause of the vision loss. In fact, associations between worse visual acuity and QoL have already been demonstrated.[7, 32] Moreover, previous work have suggested that poor educated patients might have higher levels of emotional distress (including depression, anxiety, and anger) and physical distress (including aches and pains and malaise), which could influence the responses of the QoL questionnaire.[33]

The current study has limitations. Even though Rasch analysis is becoming the gold standard for scoring patient-reported outcome measures in ophthalmology, a multilevel model that allows simultaneous analysis of different dimensions in a multidimensional instrument could also be used.[8, 10, 34] Our sample consisted of patients with cataract, glaucoma and AMD. Thus, future studies should investigate psychometric validity of the Rasch calibrated version of the NEI VFQ-25 in a sample with more varied range of eye diseases.

CONCLUSION

Our findings indicate that the Brazilian Portuguese version of the NEI VFQ-25 is not psychometrically optimal for assessing QoL related only to visual function. Rather, we found a second trait, described as a socioemotional component from results of the Rasch analysis. Thus, in order to obtain psychometrically valid constructs, both components with their respective subscales and items (visual functioning and socioemotional) should be analyzed separately. Future studies in Brazil including patients with different eye diseases are needed to substantiate our findings and evaluate the sensitivity of this calibrated version of the NEI VFQ-25.

Acknowledgements

Not applicable

REFERENCES

1. The World Health Organization Quality of Life assessment (WHOQOL): position paper from the World Health Organization. Soc Sci Med 1995, 41:1403-1409.
2. Khanna D, Tsevat J: Health-related quality of life--an introduction. Am J Manag Care 2007, 13 Suppl 9:S218-223.
3. Mangione CM, Lee PP, Pitts J, Gutierrez P, Berry S, Hays RD: Psychometric properties of the National Eye Institute Visual Function Questionnaire (NEI-VFQ). NEI-VFQ Field Test Investigators. Arch Ophthalmol 1998, 116:1496-1504.
4. Suzukamo Y, Oshika T, Yuzawa M, Tokuda Y, Tomidokoro A, Oki K, Mangione CM, Green J, Fukuhara S: Psychometric properties of the 25-item National Eye Institute Visual Function Questionnaire (NEI VFQ-25), Japanese version. Health and Quality of Life Outcomes 2005, 3:65-65.
5. Romero M, Vivas-Consuelo D, Alvis-Guzman N: Is Health Related Quality of Life (HRQoL) a valid indicator for health systems evaluation? SpringerPlus 2013, 2:664.
6. Karimi M, Brazier J: Health, Health-Related Quality of Life, and Quality of Life: What is the Difference? Pharmacoeconomics 2016, 34:645-649.
7. Mangione CM, Lee PP, Gutierrez PR, Spritzer K, Berry S, Hays RD, Investigators NEIVFQFT: Development of the 25-item National Eye Institute Visual Function Questionnaire. Arch Ophthalmol 2001, 119:1050-1058.
8. Medeiros FA, Gracitelli CP, Boer ER, Weinreb RN, Zangwill LM, Rosen PN: Longitudinal changes in quality of life and rates of progressive visual field loss in glaucoma patients. Ophthalmology 2015, 122:293-301.
9. Gracitelli CP, Abe RY, Tatham AJ, Rosen PN, Zangwill LM, Boer ER, Weinreb RN, Medeiros FA: Association Between Progressive Retinal Nerve Fiber Layer Loss and Longitudinal Change in Quality of Life in Glaucoma. JAMA Ophthalmol 201[illegible].
10. Diniz-Filho A, Abe RY, Cho HJ, Baig S, Gracitelli CP, Medeiros FA: Fast Visual Field Progression Is Associated with Depressive Symptoms in Patients with Glaucoma. Ophthalmology 2016, 123:754-759.
11. Massof RW, Fletcher DC: Evaluation of the NEI visual functioning questionnaire as an interval measure of visual ability in low vision. Vision Res 2001, 41:397-413.
12. Marella M, Pesudovs K, Keeffe JE, O'Connor PM, Rees G, Lamoureux EL: The psychometric validity of the NEI VFQ-25 for use in a low-vision population. Invest Ophthalmol Vis Sci 2010, 51:2878-2884.
13. Pesudovs K, Gothwal VK, Wright T, Lamoureux EL: Remediating serious flaws in the National Eye Institute Visual Function Questionnaire. J Cataract Refract Surg 2010, 36:718-732.
14. Andrich D: Rating scales and Rasch measurement. Expert Rev Pharmacoecon Outcomes Res 2011, 11:571-585.
15. Dougherty BE, Bullimore MA: Comparison of scoring approaches for the NEI VFQ-25 in low vision. Optom Vis Sci 2010, 87:543-548.

16. Simao LM, Lana-Peixoto MA, Araujo CR, Moreira MA, Teixeira AL: The Brazilian version of the 25-Item National Eye Institute Visual Function Questionnaire: translation, reliability and validity. Arq Bras Oftalmol 2008, 71:540-546.
17. Bradley C, Massof RW: Validating Translations of Rating Scale Questionnaires Using Rasch Analysis. Ophthalmic Epidemiol 2017, 24:1-2.
18. Chylack LT, Jr., Wolfe JK, Singer DM, Leske MC, Bullimore MA, Bailey IL, Friend J, McCarthy D, Wu SY: The Lens Opacities Classification System III. The Longitudinal Study of Cataract Study Group. Arch Ophthalmol 1993, 111:831-836.
19. Seddon JM, Sharma S, Adelman RA: Evaluation of the clinical age-related maculopathy staging system. Ophthalmology 2006, 113:260-266.
20. Coleman HR, Chan CC, Ferris FL, 3rd, Chew EY: Age-related macular degeneration. Lancet 2008, 372:1835-1845.
21. Boone W: Understanding Person Measures. In Rasch Analysis in the Human Sciences. Edited by Boone W, Staver J, Yeale M. New York: Springer; 2014: 69-92
22. Bond TG, Fox CM: Applying the Rasch Model. Fundamental Measurement in the Human Sciences. 2nd Edition edn; 2007.
23. Peters D, Heijl A, Brenner L, Bengtsson B: Visual impairment and vision-related quality of life in the Early Manifest Glaucoma Trial after 20 years of follow-up. Acta Ophthalmol 2015, 93:745-752.
24. Wright BD LJ, Gustafson JE, Martin-Lof P: Reasonable mean-square fit values. 1994, 8:370.
25. Smith EVJ: Detecting and evaluating the impact of multidimensionality using item fit statistics and principal component analysis of residuals. J Appl Meas 2002, 3:205–231.
26. Wright BD, Douglas GA: Best test design and selftailored testing. Research Memorandum. Chicago: Statistical Laboratory, Department of Education, University of Chicago; 1975.
27. Globe DR, Varma R, Torres M, Wu J, Klein R, Azen SP: Self-reported comorbidities and visual function in a population-based study: the Los Angeles Latino Eye Study. Arch Ophthalmol 2005, 123:815-821.
28. Schulze-Bonsel K, Feltgen N, Burau H, Hansen L, Bach M: Visual acuities "hand motion" and "counting fingers" can be quantified with the freiburg visual acuity test. Invest Ophthalmol Vis Sci 2006, 47:1236-1240.
29. Smith AB, Wright EP, Rush R, Stark DP, Velikova G, Selby PJ: Rasch analysis of the dimensional structure of the Hospital Anxiety and Depression Scale. Psychooncology 2006, 15:817-827.
30. Chien T-W, Wang W-C, Wang H-Y, Lin H-J: Online assessment of patients' views on hospital performances using Rasch model's KIDMAP diagram. BMC Health Services Research 2009, 9:135-135.
31. Prieto L, Alonso J, Lamarca R: Classical test theory versus Rasch analysis for quality of life questionnaire reduction. Health and Quality of Life Outcomes 2003, 1:27-27.
32. Hirneiss C: The impact of a better-seeing eye and a worse-seeing eye on vision-related quality of life. Clin Ophthalmol 2014, 8:1703-1709.

33. Ross CE, Van Willigen M: Education and the Subjective Quality of Life. Journal of Health and Social Behavior 1997, 38:275-297.
34. Gorter R, Fox J-P, Twisk JWR: Why item response theory should be used for longitudinal questionnaire data analysis in medical research. BMC Medical Research Methodology 2015, 15:55.

Fig 1 Click here to download Figure Figure 1.tif

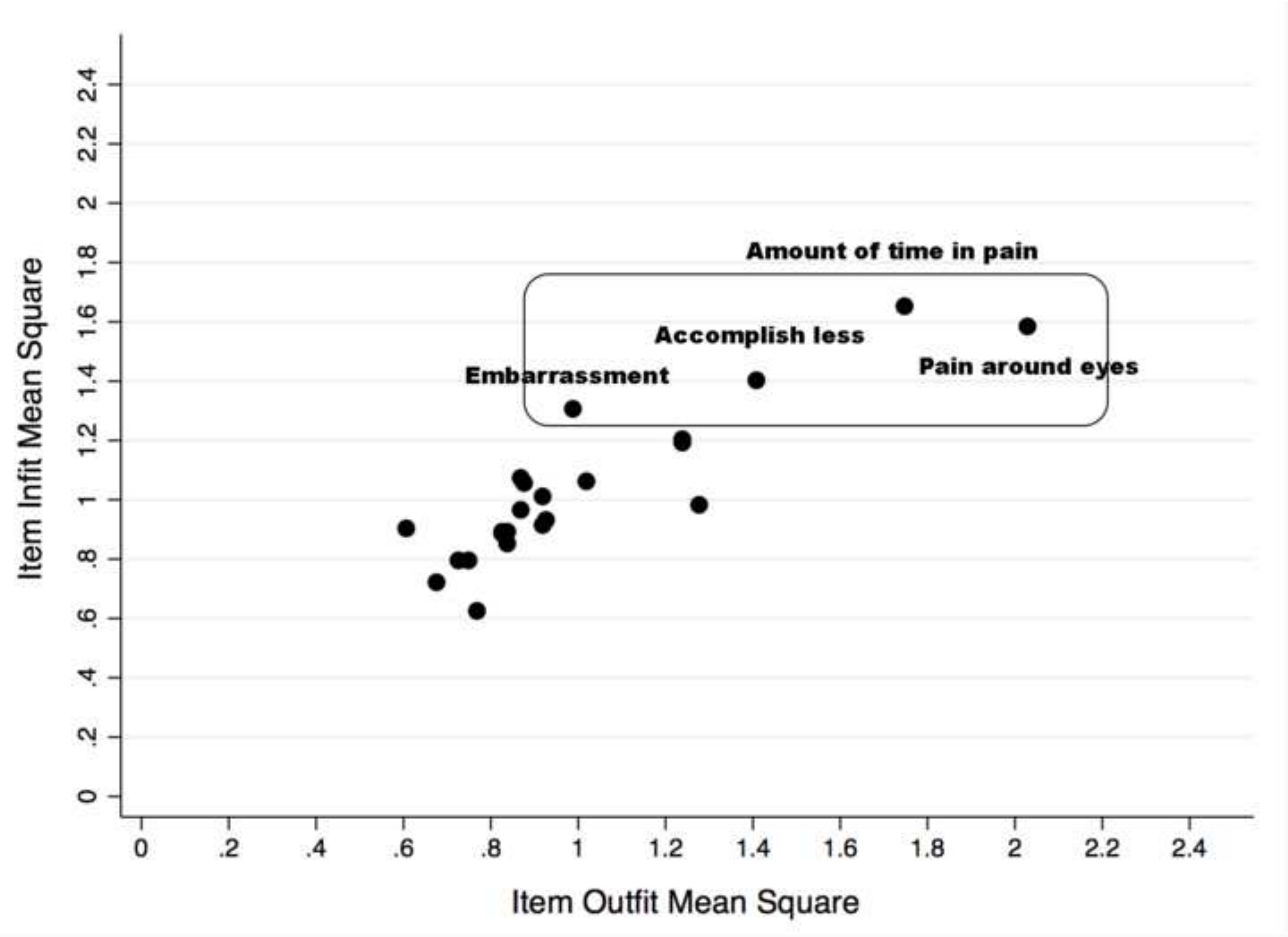

Fig 2

Click here to download Figure Figure 2 new.tif

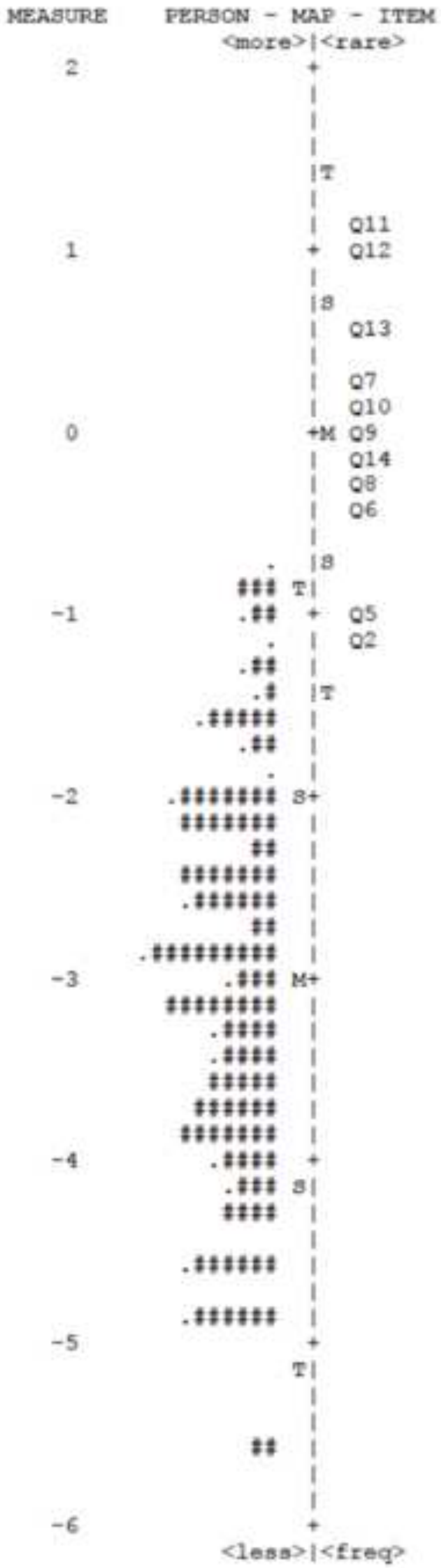

Supplement: Attachment — Submitted filename: PONE-D-19-19847_reviewer.pdf [file pone.0226086.s006.pdf]
