# Supplementary material for: Psychometric properties of the Portuguese version of the National Eye Institute Visual Function Questionnaire-25
Source: PLoS One. 2019 Dec 10;14(12):e0226086. doi: 10.1371/journal.pone.0226086 (PMC6903730; doi:10.1371/journal.pone.0226086)
Supplement: Attachment — Submitted filename: Point by Point - Plos ONE - 09.29.2019.docx [file pone.0226086.s007.docx]

**POINT-BY-POINT RESPONSE FORM**

**Manuscript #:** PONE-D-19-19847

**Manuscript title:** Psychometric Properties of the Portuguese Version of the National Eye Institute Visual Function Questionnaire-25

| Suggestion, Question,  or Comment from  Reviewer #1 | Author’s Response | Change in the Manuscript |
| --- | --- | --- |
| The authors investigate the psychometric properties of the Brazilian Portuguese version of the National Eye Institute Visual Function Questionnaire (NEI VFQ-25) in a group of patients with different eye  diseases. This is an important topic, as no study has yet applied Rasch Analysis to the Brazilian Portuguese version of the NEI VFQ-25. The study is well designed, executed and reported. However, I have a few comments. | We thank the reviewer for the comments. | N/A |
| Material and Methods: Adding some subheading could improve flow and readability for the reader. | We have made the proper changes as suggested. | We have included a subheading for Rasch Analysis |
| Not all readers are familiar with the NEI VFQ-25. Could you please provide more information about the  NEI VFQ-25 (how initially developed, how many response options, which scaling [Likert scaling?],  subscales)? | We have changed the text and figure legend as suggested. | We have included the following sentence: “The NEI VFQ was originally developed with 51 items to capture the influence of vision impairment on multiple dimensions of health related QoL, such as emotional status and social functioning. Mangione et al validated a shorter and reliable version with 25 items.” |
| Targeting: Did you inspect the person-item map and calculated the difference between item and person  means before revising the questionnaire? Knowing if the difficulty of the items adequately targets the ability  of the sample provides valuable information. | We have made the proper changes as suggested. | We have included this sentence in the Results section: “We investigate targeting using Wright person-item maps. We found that our sample did not ideally matched items for both original versions NEI VFQ-25. Even after removal of misfitting items, most of the items (right-side, top of the scale) were not able to cover people with most visual ability (left side, bottom of the scale).” |
| Figure 2: The mean person achievement measure is much lower than the mean item difficulty, suggesting  a lack of items at the low-ability end. Could you please explain and describe that? This finding is completely counter-intuitive as most questionnaires struggle with items that are too easy, in particular in questionnaires  trying to capture VRQOL. | We have made the proper changes as suggested. | We have now included the following sentence in the Discussion section: “The Wright person-item map revealed that items did not adequately covered all spectrum of visual abilities. In fact, most of uncovered percentage of patients represents persons with more visual ability (Figure 2). This may be explained by our sample did not include enough people with significant visual impairment.” |
| Table 2: Does the SAP MD values only refer to glaucoma patients or to the overall sample? | We thank the reviewer for the comments. | The SAP MD values refer only to glaucoma patients. |
| Could you please add more information on visual ability of the participants? Please report n/% of the  sample in the categories none/mild/moderate/severe visual impairment. | We thank the reviewer for the comments. | In table 2 we have the information that 24.6% of the sample had low vision in one or both eyes. |
| Line 252: The authors say, that four items were found to misfit with Infit and/or Outfit MNSQ values <0.7  or >1.3. In Table 3 Infit and Outfit MNSQ values for Q24 are within these values, so why was this item found  to misfit? Same for Q25. | We thank the reviewer for the comments. | We have reviewed the manuscript and fixed the error. “Three items (Q4, Q17, and Q19) were found to misfit (from subscales: mental health and role limitations). |
| Line 226: The authors say, that they investigated the relationship between final Rasch-calibrated NEI  VFQ-25 scores with socioeconomic variables using a linear regression model. Could you please provide the  results in a table in the supplement to see raw and adjusted effects? | We thank the reviewer for the comments. | We have now uploaded the table as requested. |
| Most of the readers might not be familiar with Rasch analysis, so please explain in more detail the factor analysis/factor loading you used to assess unidimensionality of the scale. Otherwise it might not be easy to follow why items Q1, Q3, Q21, Q22, Q17, A18, Q20 and Q 23 were excluded? | We thank the reviewer for the suggestions. | We have now included this sentence in the Discussion section to facilitate the reader’s understanding.  “The purpose of using principal component analysis and inspecting contrast plots is to find patterns in the dataset and discover groups of items that share the same patterns of unexpectedness, which could represent a secondary dimension, proving that the instrument is not unidimensional.” |
| 10. Please cite additional literature, which also reports the NEI-VFQ to be multi-dimensional. | We thank the reviewer for the suggestions. | We have now the following references regarding multidimensionality of the NEI VFQ-25:  12.Marella M, Pesudovs K, Keeffe JE, O'Connor PM, Rees G, Lamoureux EL. The psychometric validity of the NEI VFQ-25 for use in a low-vision population. Invest Ophthalmol Vis Sci. 2010;51(6):2878-84. doi: 10.1167/iovs.09-4494. PubMed PMID: 20089878.  13. Pesudovs K, Gothwal VK, Wright T, Lamoureux EL. Remediating serious flaws in the National Eye Institute Visual Function Questionnaire. J Cataract Refract Surg. 2010;36(5):718-32. doi: 10.1016/j.jcrs.2009.11.019. PubMed PMID: 20457362.  15. Kovac B, Vukosavljevic M, Djokic Kovac J, Resan M, Trajkovic G, Jankovic J, et al. Validation and cross-cultural adaptation of the National Eye Institute Visual Function Questionnaire (NEI VFQ-25) in Serbian patients. Health and quality of life outcomes. 2015;13:142-. doi: 10.1186/s12955-015-0330-5. PubMed PMID: 26370558. |
| Line 39: Typing error/ double word: “National Eye Institute Visual Questionnaire (NEI VFQ-25) questionnaire” | We thank the reviewer for the comments. | We have corrected the error. |

| **Suggestion, Question,**  **or Comment from**  **Reviewer #2** | **Author’s Response** | **Change in the Manuscript** |
| --- | --- | --- |
| The purpose of this manuscript is to investigate the psychometric properties of the Brazilian Portuguese version of the National Eye Institute Visual Function Questionnaire (NEI VFQ-25) questionnaire  in a group of patients with different eye diseases by Rasch analysis. However, The authers found that  Portuguese version of the NEI VFQ-25 is not a unidimensional instrument in measuring psychometric  properties. Although they suggest that analyzing both visual function and socio-emotional components  separately may be a valid method, they did not test the validity of this method. In addition, some mistakes  in grammar was found in the manuscript. | We thank the reviewer for the comment. | We have corrected the grammar errors in the text. |

| **Suggestion, Question,**  **or Comment from**  **Reviewer #3** | **Author’s Response** | **Change in the Manuscript** |
| --- | --- | --- |
| In this paper, Abe et al. investigated the psychometric properties of the Brazilian Portuguese  version of the National Eye Institute Visual Function Questionnaire-25. They found the Portuguese version of  NEI VFQ-25 is not a unidimensional instrument, and suggested the visual functioning and socio-emotional  components should be analyzed separately. In general, the paper is well written and in a good quality. The  study is well designed, the data are convincing, the analysis is cogent. | We thank the reviewer for the comment. | N/A |
| In the methods part, the author may want to elaborate how the NEI VFQ-25 questionnaires were  conducted. Were they self-administered or interviewer-administered? | We thank the reviewer for the suggestion. | We have included the following sentence: “The questionnaire was interview-administered.” |
| In the methods part (line 206), “Socio-economic questionnaires were also administered along with the  NEI VFQ-25 to all patients.” A sample of this questionnaire should be attached to this manuscript,  preferably as supplementary materials. | We thank the reviewer for the comment. | We have now attached the socio-economic questionnaire as supplementary material. |
| 3. In the results part (line 252), “Four items (Q4, Q19, Q24 and Q25) were found to misfit (from subscales:  general health, mental health, ocular pain and role limitations) with infit and/or outfit mean scores >1.3.”.  However, from Table 3, the infit MNSQ and outfit MNSQ of Q24 are 0.96 and 0.87 respectively, and are both  less than 1.3. | We thank the reviewer for the comment. | We have reviewed the manuscript and fixed the error. “Three items (Q4, Q17, and Q19) were found to misfit (from subscales: mental health and role limitations). |
| 4. As authors wrote in the intro (line 110-115), “Marella and colleagues have suggested that the NEI VFQ-25 questionnaire does not seem to be unidimensional, and that the questionnaire items may actually be  measuring two different underlying constructs, one related to visual functioning and another to socioemotional status.” It would be necessary for the authors to include the comparison between the findings from the present study with Marella et al.’s result. | We thank the reviewer for the comment. | In the Discussion section we have included the following sentence: “In addition, Marella et al performed a similar investigation with a group of low vision patients and also found that the NEI VFQ-25 is a better performing instrument when divided into two different scales, with items from General health (Q1), Mental Health (Q3, Q21, Q22 and Q25), Role limitations (Q18), Dependency (Q20, Q23 and Q24) belonging to a secondary dimension, corroborating the findings of our study. [12]” |
| 5. It is surprising to see that the items authors found misfitting in this study (namely Q4, Q19, Q24 and Q25) were very much different from the misfitting questions Pesudovs et al. found in their study (PMID: 20457362). Authors need to provide explanations for this difference. | We thank the reviewer for the comment. | The work from Pesudovs included only patients with cataract. Our sample included also patients with glaucoma and AMD. The variety of eye diseases in our sample might have influenced the results when evaluating fit performance. |

| **Suggestion, Question,**  **or Comment from**  **Reviewer #4** | **Author’s Response** | **Change in the Manuscript** |
| --- | --- | --- |
| This is an interesting finding. The authors investigated the psychometric properties of NEI VFQ-25 using Rasch analysis in a population of Brazilian patients with a variety of eye diseases. They found that the Brazilian Portuguese version of the NEI VFQ-25 is not psychometrically optimal for assessing QoL related only to visual function. Moreover, they also observed a second trait, described as a socioemotional component from results of the Rasch analysis. Overall, the authors have described the experimental design and knowledge gaps to be generally filled with this study. The results have been generally presented in a  reasonable and expected approach with solid statistical analysis. | We thank the reviewer for the comment. | N/A |
