# Supplementary material for: Psychometric properties of the Portuguese version of the National Eye Institute Visual Function Questionnaire-25
Source: PLoS One. 2019 Dec 10;14(12):e0226086. doi: 10.1371/journal.pone.0226086 (PMC6903730; doi:10.1371/journal.pone.0226086)
Supplement: Attachment — Submitted filename: Point by Point - Plos ONE - November 2019.docx [file pone.0226086.s009.docx]

**POINT-BY-POINT RESPONSE FORM**

**Manuscript #:** PONE-D-19-19847

**Manuscript title:** Psychometric Properties of the Portuguese Version of the National Eye Institute Visual Function Questionnaire-25

| Suggestion, Question,  or Comment from  Reviewer #2 | Author’s Response | Change in the Manuscript |
| --- | --- | --- |
| In the revised manuscript, the authors mentioned that three items were found to misfit according to Rasch analysis and mental health was cancelled, the reason should be addressed. | We thank the reviewer for the comments. We had to run new analysis to double check for possible errors. Please see supplemental file (number 1) to see the complete table for FIT statistics. | Line 251 we have replaced for the following sentence: “Results of Rasch analysis are shown in Table 3. Five items (Q4, Q11, Q17, Q19 and Q25) were found to misfit (from subscales: social function, mental health, ocular pain and role limitations) with infit and/or outfit mean scores >1.3 and <0.7.” |
| Secondly, nine items Instead of eight ones loaded positively onto the first contrast with a correlation higher than 0.4 in the revised manuscript, why?. | We thank the reviewer for the comments. We had to run new analysis to double check for possible errors. Please see supplemental file (number 2 and 3) to see the complete table for summary statistics and respective loadings. | Line 278 we have replaced for the following sentences:  “For the current work, the principal component analysis of the residuals showed that the variance explained by the principal component was comparable for empirical calculation (55.5%) and by the model (56.1%). This suggests that the questionnaire was not unidimensional. Moreover, the unexplained variance explained by the first contrast was 2.81 eigenvalue units and the second contrast was 2.04 eigenvalue units with no further contrasts exceeding 2.0 eigenvalue units. These findings suggested the presence of a second dimension in the scale. Eight items loaded (correlation>0.4) positively onto the first contrast and belonged to: near vision (Q5), distance vision (Q8 and Q14), mental health (Q21and Q22) and dependency (Q20, Q23 and Q24).” |
| Thirdly, they did not test the validity of both visual function and socio-emotional components separately. | We thank the reviewer for the comments | In Table 5 we have reported the Rasch Analysis Fit Statistics for the Visual Function and Socioemotional Components separately |
| Finally, some results in revised manuscript is different from the first ones, does it means that the consistency of psychometric properties of the Brazilian Portuguese version of the National Eye Institute Visual Function Questionnaire (NEI VFQ-25) questionnaire in a group of patients with different eye diseases by Rasch analysis is not same? | We thank the reviewer for the comments. We had to run new analysis to double check for possible errors. By doing this step, we do not believe that consistency was weakened. Instead, we believe that after successive reviews, now the paper show reliable and relevant data regarding psychometric properties of the Brazilian Portuguese version of the National Eye Institute Visual Function Questionnaire (NEI VFQ-25). | N/A |
